# Annelid genomes: *Enchytraeus crypticus*, a soil model for the innate (and primed) immune system

**DOI:** 10.1038/s41684-021-00831-x

**Published:** 2021-09-06

**Authors:** Mónica J. B. Amorim, Yannick Gansemans, Susana I. L. Gomes, Filip Van Nieuwerburgh, Janeck J. Scott-Fordsmand

**Affiliations:** 1 Department of Biology & CESAM, University of Aveiro, Aveiro, Portugal; 2 Department of Pharmaceutics, Laboratory of Pharmaceutical Biotechnology, Ghent University, Ghent, Belgium; 3 Department of Biosciences, Aarhus University, Silkeborg, Denmark

**Keywords:** Genomics, Environmental sciences

## Abstract

Enchytraeids (Annelida) are soil invertebrates with worldwide distribution that have served as ecotoxicology models for over 20 years. We present the first high-quality reference genome of *Enchytraeus crypticus*, assembled from a combination of Pacific Bioscience single-molecule real-time and Illumina sequencing platforms as a 525.2 Mbp genome (910 gapless scaffolds and 18,452 genes). We highlight isopenicillin, acquired by horizontal gene transfer and conferring antibiotic function. Significant gene family expansions associated with regeneration (long interspersed nuclear elements), the innate immune system (tripartite motif-containing protein) and response to stress (cytochrome P450) were identified. The ACE (Angiotensin-converting enzyme) — a homolog of ACE2, which is involved in the coronavirus SARS-CoV-2 cell entry — is also present in *E. crypticus*. There is an obvious potential of using *E. crypticus* as a model to study interactions between regeneration, the innate immune system and aging-dependent decline.

## Abstract (web summary)

*Enchytraeus crypticus* is a soil-dwelling annelid worm that has been used over the past two decades as an ecotoxicology model. Here, Mónica Amorim and colleagues present the first genome for *E. crypticus*. The authors identify a number of expanded gene families, including several involved with innate immunity.

## Main

As Charles Darwin pointed out well over 100 years ago, ‘The plough is one of the most ancient and valuable of man’s inventions; but long before he existed the land was in fact regularly ploughed, and still continues to be thus ploughed by earthworms. It may be doubted whether there are many other animals which have played so important a part in the history of the world, as have these lowly organized creatures.’^1^. Without worms, it is likely that the earth’s soil would not be capable of sustaining the growth of food for humans and other plant-eating species. Annelid worms cover >22,000 species and are found worldwide in all types of habitats. Yet, knowledge of their genome was virtually absent until now. Sequencing big animals (e.g., gorillas^2^) has a large impact for conservation, but the importance of small species, often invisible at the naked-eye scale, is well known for their role in supporting life itself^3,4^.

Well-known model species with sequenced genomes include *Drosophila melanogaster*, *Caenorhabditis elegans* and *Arabidopsis thaliana*. However, very few ecotoxicology models have become genome model species, i.e., equipped with genomic-level endpoints in addition to phenotypic endpoints. The genome of *Folsomia candida*, a standard terrestrial ecotoxicology arthropod collembolan species^5^, was sequenced^6^ in 2017. This added on to the first aquatic ecotoxicology Daphnid model^7^. The species *Daphnia pulex* was sequenced^8^ in 2008; however, this is not the commonly tested *Daphnia magna* species. Recently among soil annelids, the *Eisenia andrei* genome has been sequenced^9^; this provides a high-quality assembly for a soil representative model that is also used in ecotoxicology similarly to *Eisenia fetida*. Other sequenced annelids include *Helobdella robusta* and *Capitella teleta*, but these are not ecotoxicology models.

**Fig. 1:**
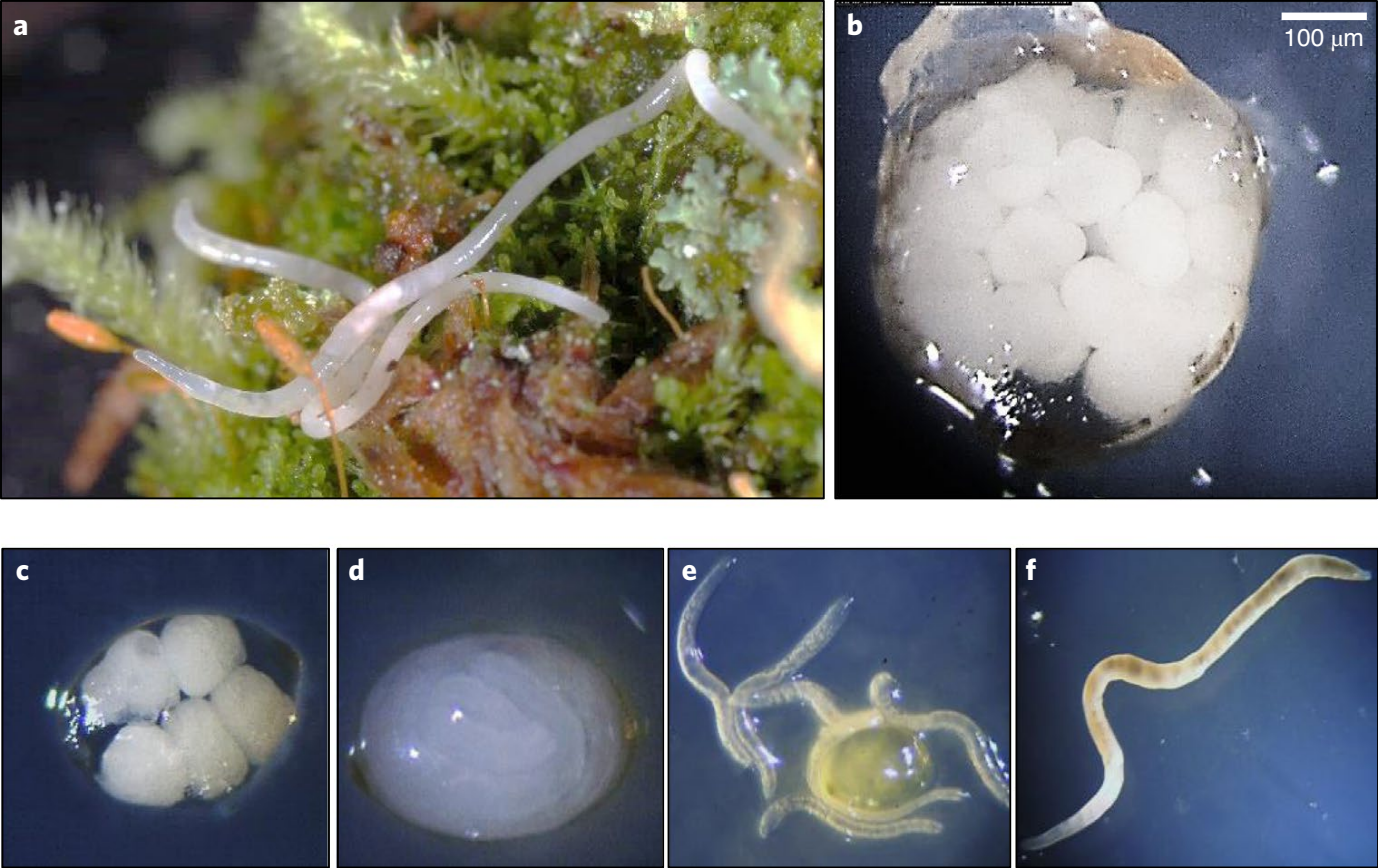
*Enchytraeus crypticus* (Annelida: Enchytraeidae). *E. crypticus* are soil invertebrates, belonging to the Oligochaete. Their size ranges from 6 to 9 mm, and they reproduce both sexually and asexually, carrying the cocoons with the embryos in the clitellum and releasing these when matured; they are semi-transparent, and the cocoons and other organelles can be visualized directly (e.g., under a binocular in the culture dishes). **a**, A photo in a natural habitat assembly. **b**, A cocoon with embryos. **c**, A cocoon at post-eggs stage (start of differentiation). **d**, A cocoon with juveniles. **e**, Juveniles from a hatched cocoon; **f**, An adult.

The species sequenced in this study, *Enchytraeus crypticus*, is a soil invertebrate belonging to the phylum Annelida, class Clitellata, order Oligochaeta and family *Enchytraeidae* (Fig. 1). Enchytraeids are the most important organisms in many habitats, dominant both in biomass and abundance^10^ and ranging between 10^2^ and 10^5^ individuals/m^2^. They belong to the saprophagous mesofauna and play an important role in the degradation of organic matter. Contrary to many larger earthworms, which live in the humus or soil surface, enchytraeids inhabit the actual soil layer. Through their feeding activity, the soil assumes a fine-grained ‘crumb’ structure with an often higher stability than that of the bulk soil^11^. Enchytraeids are generally obligatory amphimictic hermaphrodites, but some species are able to reproduce by either parthenogenesis or self-fertilization. Most species reproduce sexually by means of egg and sperm production, cross-fertilization and cocoon deposition. *E. crypticus* can also reproduce via fragmentation: observations confirmed the regenerative ability in the posterior part (tail segments) after artificial amputation, whereas the anterior part was not able to regenerate^12^. One hypothesis is that autotomy can be used by this species as a self-defense mechanism in response to stress or injuries from physical or chemical stimuli, allowing detoxification and survival. *Enchytraeus crypticus* are probably diploid, although this has not been confirmed.

Because of their relevance and sensitivity, enchytraeids are standard models when it comes to evaluating the environmental risk of human-made compounds^13^ and have been used for >20 years for hazard assessment of chemicals. There are standardized protocols to assess survival and reproduction (ISO (International Standard Organization) and OECD (Organization for Economical Cooperation and Development))^14^, bioaccumulation^15–17^ and avoidance^18^ in enchytraeids, as well as vast arrays of other endpoints available.

There are few terrestrial environmental species with such a tool suite covering genotypic to phenotypic endpoints that are also ecotoxicological models. There has been impressive development in terms of molecular tools for *E. crypticus*, with a full transcriptome^19^ and suite of omics tools available at present; these include customized microarrays with a wide range of transcriptomics applications^20–26^, proteomics^27^, metabolomics^28^ and epigenetics^29,30^, with considerations of big data analysis and progress^24^. This ecotoxicology model species also includes phenotype-level endpoints for embryotoxicity^31^, full life cycle with hatching success, growth, maturation, survival, reproduction^32–35^, multigeneration^36,37^, full life span^38^, species interactions by using multispecies test systems^39–42^, histological tools^43^, oxidative stress biomarkers^44–47^ and cellular energy allocation^48,49^. The possibility of studying embryo development (and all life stages in the full life cycle test) in *E. crypticus* and its ability to reproduce via regeneration^12^ also represents some major opportunities. Hence, the progress toward sequencing the genome of this species will provide a major step forward in many related fields (e.g., for evolutionary studies and understanding the mechanisms underlying stress responses).

In this study, we present the first reference genome of *E. crypticus*, assembled from a combination of long and short reads produced on the Pacific Bioscience single-molecule real-time (SMRT) and Illumina sequencing platforms.

## Results

### De novo assembly and annotation of the *E. crypticus* genome

De novo assembly of the *E. crypticus* genome was done with 1.3 × 10^9^ Illumina paired-end reads, 1.3 × 10^8^ Illumina mate-pair reads and 1.2 × 10^6^ PacBio long reads. These were assembled into 910 gapless scaffolds ≥1,000 nt long, for a total of 525.2 Mbp having an N50 of 1.2 Mbp and an L50 of 118 (see Table 1 for a summary). The largest scaffold had a sequence length of 5.7 Mbp. The GC content of the genome was 35.4%. Genome quality and completeness were checked via a benchmarking universal single copy orthologs (BUSCO) analysis: out of 954 metazoan genes, the method detected 856 (89.7%) complete single-copy orthologs and 41 (4.3%) complete but duplicated orthologs. There were 14 (1.5%) fragmented and 43 (4.5%) missing orthologs. Finally, 97.7% of the Illumina input reads and 80.6% of the PacBio reads mapped back on the genome.

**Table 1:** *E. crypticus* genome properties

De novo assembly	
Number of scaffolds	910
Total genome size (bp)	525,192,231
Largest scaffold (bp)	5,688,427
Smallest scaffold (bp)	1,352
N50 (bp)	1,254,661
L50	118
GC (%)	35.41
Percent Illumina reads mapping to the genome	97.7
Percent PacBio reads mapping on the genome	80.6
Average coverage depth	350
Mitochondrial genome size (bp)	15,205
Genome structure	
Total number of genes	18,452
Genes as genome fraction (%)	24.78
Average gene length (bp)	7,054
Number of protein-coding genes	16,424
Protein-coding genes as genome fraction (%)	24.70
Exons as genome fraction (%)	5.04
Introns as genome fraction (%)	19.68
Repeats as genome fraction (%)	39.03
Functional annotation	
Number of genes with putative functions	13,010
Number of genes with Gene Ontology terms	7,540
Number of genes with InterPro domain information	11,468
Validation	
Complete BUSCOs (%)	94.00
Detected BUSCOs (complete + partial) (%)	95.50

Supported by experimental data (see Methods), the genome was predicted to contain a total of 18,452 gene models, accounting for 24.78% of the genome size and a gene density of 35 genes per mega base pair. We found 16,424 protein-coding genes, of which 82.8% were supported by public transcriptome data. The identified non-coding RNA genes consisted of 295 rRNA genes, 815 tRNA genes and 918 tRNA pseudogenes. A list of the predicted *E. crypticus* gene models is presented in Supplementary Table 1 (complemented by the genome, found in the Supplementary Data).

Repeated DNA segments comprised 39.03% of the genome and consisted, in decreasing order of abundance, of long interspersed nuclear elements (LINE), unclassified repeats, DNA transposons and simple repeats (Supplementary Table 2).

**Fig. 2:**
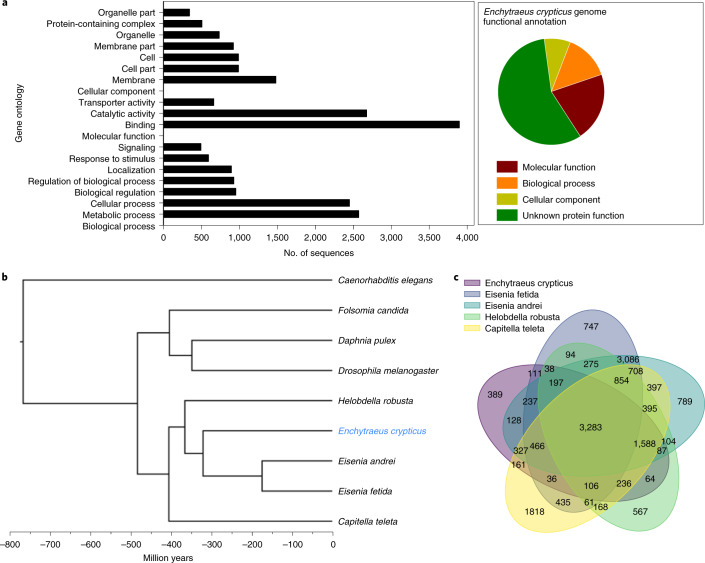
*Enchytraeus crypticus* genome. **a**, GO (Gene Ontology) functional distribution, including the top 50 per biological process, molecular function and cellular component. **b**, Ultrametric phylogenetic tree based on orthogroup analysis from this study. The indicative age of the root was derived from the TimeTree database divergence time of *C. elegans* to all members of the clade composed by *F. candida, D. pulex* and *D. melanogaster*. **c**, Comparative analysis via Venn diagram, displaying the number of shared gene families between Annelids *(E. crypticus, E. fetida, E. andrei, H. robusta* and *C. teleta*) as determined by OrthoFinder.

From the 7,540 genes with gene ontology annotation, the distribution showed a majority of genes involved in molecular functions, followed by biological processes and cellular components (Fig. 2a).

Most of the genes are involved in binding and catalytic activity within molecular functions, while for biological processes, metabolic and cellular processes are the most represented, followed by regulation, response to stimulus and signaling. Further detail on each gene ontology (GO) term can be found in Supplementary Fig. 1.

### De novo assembly and annotation of the *E. crypticus* mitochondrial genome

Because the full genome assembly did not contain a scaffold representing an intact mitochondrial genome, a separate assembly was attempted by using the Illumina paired-end reads only and specialized software. The resulting mtDNA of *E. crypticus* has a length of 15,205 bp. When searching for this sequence in the main genome assembly, two scaffolds containing fragmented copies of the mitochondrial genome were identified and removed from the assembly. Annotation of the mitochondrial genome detected a replication origin, 22 tRNA genes, 2 rRNA genes and 13 protein-coding genes, for a total of 37 genes (see MT (Mitochondrial) scaffold in Supplementary Table 1). The gene order is identical to that reported for *Lumbricus terrestris*^50^, with the exception of a non-coding segment located between *trnH* and *nad5* instead of separating *trnR* from *trnH*. A map of the annotated mitochondrial genome is available in Supplementary Fig. 2.

### Gene family analysis and orthogroups

The comparison between *E. crypticus* and eight other relevant species assigned 218,791 genes to orthogroups (~85% (>80%)) (Supplementary Table 3). A phylogenetic tree based on the orthogroup analysis is shown in Fig. 2b. The number of shared orthogroups between the four annelid species is represented in the Venn diagram (Fig. 2c). One would expect larger overlap between *E. fetida* and *E. andrei*, but *E. fetida* data are derived from a poor-quality assembly, and hence results can change substantially once quality increases.

The list of significant expansions of gene families in *E. crypticus*, based on the z-scores, can be found in Supplementary Table 4 (see Supplementary Table 5 for the *E. crypticus* orthogroup protein description list). A total of 1,751 gene families were shared between *E. crypticus* and all the other eight species, with 104 being expanded in the *E. crypticus* genome (when including at least three more species in the comparison). The top 10 largest expansions (Supplementary Table 6) included long interspersed nuclear elements (LINEs) (129 genes), cytochrome P450 (44 genes), tripartite motif (TRIM) (26 genes), ankyrin (ANK; 19 genes), heat shock protein (16 genes), purple acid phosphatase (15 genes), paramyosin (11 genes), vitellogenin (VTG; 10 genes), caspase (10 genes) and hydroxy acid oxidase (HAO) (9 genes), besides several groups with unknown function (e.g., 54 genes in OG0000259). Other gene groups, such as inositol phosphate synthase (21 genes), potassium voltage-gated channel protein Shaw (11 genes) or nAChRbeta1:acetylcholine receptor subunit beta-like (10 genes) also showed high representation and are briefly integrated in the discussion.

With respect to lineage-specific gene families, we counted 307 orthogroups containing 1,370 genes unique for *E. crypticus* when compared to the eight selected species. An overview of the *E. crypticus*–specific orthogroups and their gene content can be found in Supplementary Table 7. Zinc fingers, one of the most abundant groups of proteins known for their wide range of molecular functions (transcriptional regulation, ubiquitin-mediated protein degradation, signal transduction, actin targeting, DNA repair, cell migration, etc.)^51^, were among the most represented. Another example included the sarcoplasmic calcium-binding protein, an invertebrate EF-hand calcium-buffering protein, suggested to have a similar function in muscle relaxation as vertebrate parvalbumin^52^.

### Collinearity analysis

**Fig. 3:**
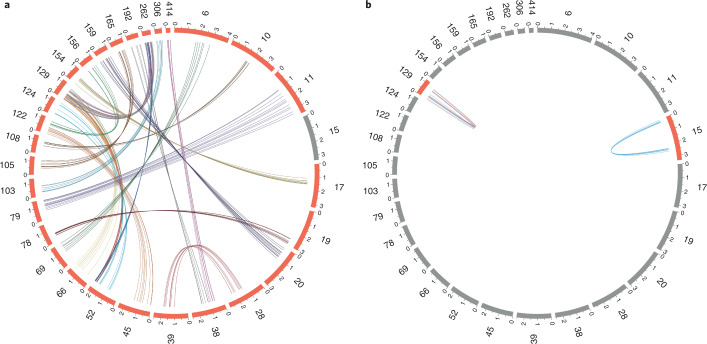
Synteny plot showing intra- and inter-scaffold collinear genes in the *E. crypticus* genome. Scaffolds were renumbered after ordering them by decreasing length for readability; only relevant scaffolds are shown. Gray bars represent scaffolds with no collinearity in the context shown. Scale in million bp. **a**, Inter-scaffold collinearity. **b**, Intra-scaffold collinearity; palindromes are drawn with blue links, tandem repeats with red links.

Intragenomic collinearity analysis detected 313 collinear genes in 25 syntenic blocks (see Supplementary Table 8 for a detailed list). Of those, one appears as an intra-scaffold palindrome on scaffold 15 (ANK) and another one as a tandem repeat in scaffold 129 (zinc finger) (Fig. 3).

### Biosynthetic gene clusters

We used antiSMASH (v5.1.2) to identify biosynthetic gene clusters in the *E. crypticus* genome. The tool reports only one multi-gene cluster as a chemical hybrid of type I polyketide synthase and non-ribosomal peptide synthetase. The two genes in this cluster were also identified as horizontal gene transfer (HGT) candidates (ECRY_011785-RA, ECRY_011786-RA and malonyl CoA-acyl carrier protein transacylase, from the fatty acid biosynthesis) although not confirmed as HGT.

### Hox genes

Based on similarity with Uniprot and HomeoDB, we identified a total 160 homeobox genes in the *E. crypticus* genome. Of these, 38 are members of the ANTP/HOXL class, which is involved in embryonic development. This number is comparable to that found in the recently assembled high-quality genome of *Metaphire vulgaris*^53^, another annelid. Supplementary Fig. 3 shows the distribution of the homeobox genes over the known classes. A complete list of identified hox genes is presented in Supplementary Table 9. Manual assessment of synteny reveals that genes of the ANTP/HOXL class exist as multiple homologs located on several scaffolds. We do, however, notice a cluster of *Hox1*, *Hox3*, two *Hox5* variants and a *Hox7* gene on scaffold scf7180000023640.912933. A smaller cluster consisting of *Hox1*, *Hox5* and *Hox7* is present on another scaffold, scf7180000023512.337295. In both cases, the orientation is the same for all genes in the cluster.

### HGT

**Fig. 4:**
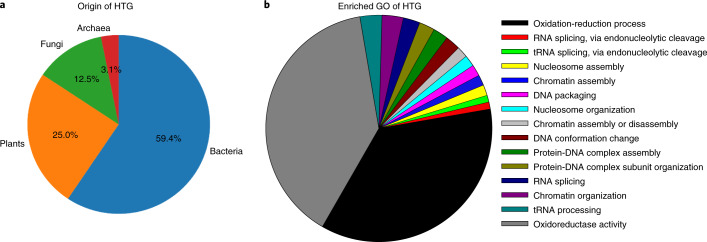
Horizontally transferred genes (HTG) in *Enchytraeus crypticus*. **a**, Origin. **b**, Enriched Gene Ontology. Supplementary Table 11 contains the *P* values for the GO terms.

By calculation of h-scores, 105 HGT candidates were initially identified; 33 of them were rejected because of the absence of native neighbor genes and long read linkage. Based on their low metazoan bitscore, five genes were confirmed to have been the result of HGT. The remaining 67 HGT candidates were subjected to a phylogenetic test and resulted in an additional 27 confirmed HGT genes, for a total of 32 genes (Supplementary Table 10). The origin of the confirmed HGT genes is represented in Fig. 4. Bacterial origin is detected for 59.4% of the HGT genes, followed by plants and fungi for 25.0% and 12.5%, respectively, and finally Archaea for 3.1%. A Gene Ontology (GO) term enrichment analysis on the set of horizontally transferred genes yields 14 Biological Process (BP) terms and a single Molecular Function term (Supplementary Table 11).

## Discussion

### Genome assembly

In this study, we produced the first high-quality genome for the oligochaete enchytraeid *E. crypticus*. The presented genome has good contiguity and completeness, as revealed by an N50 of 1.2 Mbp and an overall BUSCO score of 94.0%. The genome sequence, together with the currently over 18,000 identified genes, will allow exploration of the mechanisms underlying interactions with the worms’ environment and its potential toxicants, organ development/regeneration, adaptation and evolutionary aspects.

### Genome size

With a 525-Mbp genome, it is interesting to realize the size differences compared to other annelids: larger than the clitellate *H. robusta* (235 Mbp) and the polychaete *C. teleta* (333 Mb), but smaller than *E. andrei* (1,300 Mbp), *E. fetida* (1,000 Mbp) and *Mesenchytraeus solifugus* (1,250 Mbp)^54^. In invertebrates, genome size differences have been found to correlate with, for example, life cycle duration^55^ and negatively with developmental rate; that is, species with multiple generations per year have smaller genomes (C-values) compared to species with one generation per year.

When comparing the two enchytraeid species, the ice worm *M. solifugus* and *E. crypticus* (1,250 versus 525 Mbp), the former’s twofold larger genome size can be due to fast mutational mechanisms or to natural selection. *M. solifugus*, a small and heavily pigmented enchytraeid, inhabits glacier areas in some of the coldest and highest UV radiation habitats on earth; it also has a much longer life span, living ~10 years, compared to ~1 year for *E. crypticus*^38^.

The enchytraeid family has an interesting trait regarding freeze tolerance: an RNAseq study in *Enchytraeus albidus* showed how the population from Greenland has specific transcriptional differences compared to the German population; both of these strains are freeze tolerant, but the Greenland population is extremely freeze tolerant^56^. The involved key processes are anion transport in the hemolymph, fatty acid metabolism, metabolism and transport of cryoprotective sugars as well as protection against oxidative stress, with peroxisome and toll-like receptor (TLR) signaling pathways being differentially expressed^56^. *E. crypticus* may be a well-adapted species for its life in the deeper soil layer, more buffered from variations compared to the upper layer, where other annelids, such as *E. fetida* and *E. andrei* (compost worms) or *M. solifugus* (ice worm), live. The fact that *E. crypticus* inhabits a less-variable environment than other worms may have reduced its gene bank source for adaptation (e.g., gene duplication) to cope with a changing environment.

Species-specific evolution and environment contribute to the end result of genome size and gene diversity. For instance, the *E. crypticus* genome (life span: 12 months; size: 6–9 mm; and genome size: 525 Mb) is twice as large as that for *F. candida* (life span: 5–8 months; size: 1–5 mm; and genome size: 220 Mb), a terrestrial arthropod, but the latter has ~10,000 more genes. Among other main differences, the arthropod *F. candida* is a parthenogenetic species, whereas the oligochaete *E. crypticus* mostly reproduces sexually, besides alternatives like regeneration. For the small crustacean *Daphnia pulex* (life span: 4–6 months; length: 1–5 mm; and genome size: ~200 Mb), gene duplication seems to be at the core of their evolutionary strategy^8^. Although there seems to be a trend, a larger number of genomes would be needed to allow such an analysis.

### Collinearity

As mentioned, the arthropod *F. candida* is a parthenogenetic species, whereas the oligochaete *E. crypticus* mostly reproduces sexually. *E. crypticus* showed 313 collinear genes in 25 syntenic blocks, much less than the collembolan *F. candida* with 883 genes in 55 syntenic blocks^6^. Gene collinearity has been associated with parthenogenic reproduction types; for example, the sexually reproducing collembolan *Orchesella cincta* does not show this pattern, and the parthenogenetic nematode *Meloidogyne incognita* has a mitotic cell division reproduction system^57^. Of the few intra-collinear genes in *E. crypticus*, zinc finger appears as a tandem repeat in scaffold 129 and is also found among the lineage-specific gene families, adding to its relevance. Zinc fingers, which can have many functions (e.g., binding DNA and RNA and being involved in transcriptional regulation), have also been found in *F. candida* in a palindrome^6^. In *E. crypticus*, scaffold 129 has 60 zinc finger genes in inter-collinearity with scaffold 52, besides the 7 intra-collinear tandem repeats.

Furthermore, ANK genes are present in an intra-scaffold palindrome, together with the protocadherin FAT4 and serine/threonine-protein kinase pak-1 (see the discussion section on ANK).

### Expanded gene families and HGT

#### LINEs

Focusing on the gene family expansions, LINEs are a group of non–long terminal repeat retrotransposons that are widespread in the genome of many eukaryotes. These transposable elements are believed to contribute to the instability and evolution of genomes and are tightly regulated by DNA methylation, histone modifications and PIWI-interacting RNAs (piRNA). LINE2 has significant expansion (7.49%) in the earthworm *E. andrei* compared to other representative metazoan species: 2.52% in *C. teleta*, 3.90% in *H. robusta*, 0.00% in *Macrostomum lignano* and 0.84% in *Apostichopus japonicus*; the closely related species *E. fetida* also has a relatively high LINE2 proportion of ~4.10%^9^. With 5.04% LINE2, *E. crypticus* is closest to *H. robusta* and *E. andrei*, the top percentage species.

For *E. andrei*, the LINE2 transposable elements and gene families were functionally related to regeneration (e.g., epidermal growth factor receptor); thus, LINE2 is potentially involved in regulating genes involved in regeneration. *E. crypticus* is known to regenerate^12^, although only its posterior end, whereas *E. andrei* regenerates both. Regeneration has not yet been studied at the genomic level for *E. crypticus*.

Like regeneration, embryogenesis is a stage of high cell proliferation; this has been studied at the transcriptomic level in *E. crypticus* embryos when exposed to cadmium (Cd)^22^. The down-regulation of *pms1*, a gene coding for a protein involved in DNA mismatch repair, was observed, suggesting that Cd affects DNA synthesis and repair in *E. crypticus* embryos. Cd also induced the down-regulation of several genes involved in cell cycle/cell division, including cell division cycle proteins and cell division protein kinases. Injured *E. fetida* showed wound-induced transcriptional activation of early growth response protein 1 gene (EGR1)^9^; this could also be the case with *E. crypticus*. The epidermal growth factor receptor is a transmembrane receptor with tyrosine kinase activity that can regulate cell proliferation and differentiation. Hence, some of the mechanisms involved in regeneration also occur during embryogenesis, which is not surprising given the need for cell proliferation and differentiation in both events. Because earthworms are considered of great interest from the perspective of regenerative biology^9^, this can now be complemented by studies in *E. crypticus*; that is, the underlying mechanisms for regeneration^58^ can now also be studied in enchytraeids (*E. crypticus)*, which have a shorter life cycle than *E. andrei*.

#### TRIM

Recent studies have revealed that TRIM proteins play key roles in innate antiviral immunity. TRIM, expanded in *E. crypticus*, is a protein super-family conserved in metazoans that expanded rapidly during vertebrate evolution. There are more members in humans (65) and mice (64) than in worms (~20) and flies (<10). Many TRIM proteins are induced by type I and II interferons, which are crucial for resistance to pathogens, and several are known to be required for the restriction of infection by lentiviruses^59^.

Type I interferon induction is a central event of the immune response against viral infection, relying on the recognition of pathogens by cellular pattern recognition receptors (PRRs), which then trigger several signaling cascades resulting in pro-inflammatory cytokines and interferon production^60^. TRIM proteins are essential and act as restriction factors or by modulating PRR signaling. TLRs and other PRRs are engaged by bacterial, viral or fungal components, which triggers the innate immune responses. Although TRIM genes clearly arise from a common ancestral gene, they evolved independently, having acquired species-specific functions^59^.

Invertebrates are exposed to a wide array of natural and anthropogenic threats with which the immune system has to deal. For instance, *M. solifugus* tolerate huge amounts of UV radiation compared to many other organisms to endure in the arctic ice and snow. Melanin synthesis, which gives *M. solifugus* its dark brown color, is known to be a central mechanism of innate immunity and a major response to various immune challenges, including UV. Part of the melanin synthesis pathway is catalyzed by the enzyme phenoloxidase; the phenoloxidase cascade produces melanin and induces multiple potent bioactive agents, such as peroxinectin and Reactive Oxigen Species (ROS), that aid in phagocytosis and cell adhesion. *E. crypticus*, which has a milky transparent dermis, would have to cope with UV in a different manner.

Other examples include exposure to nanomaterial (NM) contamination, which also activates the innate immune system via different mechanisms^20,25,26^. NM recognition can first occur upon interaction with surface receptors—typically innate immune PRRs^61^. As NMs enter a biological environment, they become covered with a corona of proteins, sugars or other compounds. The coronas can mask the NM surface and prevent immune recognition. The importance of protein corona composition for NM recognition was studied in coelomocytes by using coelomic proteins (native repertoire) of the earthworm *E. fetida* compared to FBS (non-native reference)^62^. Over time, silver (Ag) NMs can competitively acquire a biological identity native to the cells in situ, although significantly greater cellular accumulation is observed with coelomic protein corona complexes, with lysenin having a key role. On the basis of the genome sequence, we can now look for similarities between *E. crypticus* and *E. fetida*, and we find that lysenin is present only in *E. fetida*. This is a case of species-specific formation of biomolecular coronas and suggests that the use of representative species may need careful consideration in assessing the risks associated with NMs. With our knowledge of the genome, it also means that a protein with similar function in *E. crypticus* can potentially be identified and possibly linked to the same initiating event.

As mentioned, various organisms may activate similar but not identical mechanisms for the recognition of and response to NMs. For instance, plant PRRs, similar to animal TLR, recognize microbe- or pathogen-associated molecular patterns and trigger defense responses (e.g., ROS production, Mitogen Activated Protein (MAP) kinase activation and induction of defense genes). Worms (e.g., both *E. fetida* and *E. crypticus*) have a wide range of genes coding for extracellular recognition proteins (e.g., lectins, peptidoglycan-recognition proteins, lipopolysaccharide- and β1,3-glucan–binding proteins and fibrinogen-related proteins), and any of these are good candidates for similar function identified for lysenin, for example, in enchytraeids.

Research in innate and primed immunity in *E. crypticus* may open new horizons for developing strategies to prevent or combat infectious diseases, inflammatory conditions and autoimmune disorders. The severe acute respiratory syndrome coronavirus 2 (SARS-CoV-2) is a compelling case of innate immune hyperactivity^63^, causing acute respiratory distress syndrome. SARS-CoV-2 enters cells through the angiotensin-converting receptor (ACE) 2 (ACE-2) receptor, which is expressed in a small set of alveolar type 2 epithelial cells. The gene coding for ACE is present in the *E. crypticus* genome, further confirming the potential of this species for immunology studies. Transcriptomic studies showed the activation of the ACE gene in *E. crypticus* as a response to stress when exposed to TiO_2_ NMs^21^ and Ag NMs^25^. This activation was material specific; for example, the Joint Research Centre (JRC) reference TiO_2_ NMs NM105, NM104 and NM103 caused an ACE upregulation when exposed under UVB light, whereas exposure to a TiO_2_-Fe–doped library without UV caused its downregulation. Ag materials caused upregulation of ACE for exposure to AgNM and Ag-PVP coated^25^. As mentioned, it has been shown that NMs are handled as invaders by cells, like viruses, and can activate similar mechanisms^64^. A related observation is that SARS-CoV-2 disables interferons—‘strikingly depressed interferon activity and elevated chemokines in individuals whose disease became severe and critical’—hence, a dosage of synthetic interferons to both healthy and infected people might help tame the disease^65^. Although there is more to coronavirus disease than innate response, *E. crypticus* may be useful for studying fundamental mechanisms (see the earlier discussion on interferons associated with the TRIM).

#### Caspases

Another expanded family in *E. crypticus* includes caspases, a group of cysteine-based proteases that are essential not only during apoptosis but also for the immune system. The role of caspases in cell death has further revealed a caspase-driven compensatory proliferation, apoptosis-induced proliferation^66^, known to be involved for some forms of regeneration (as discussed above). Several NMs activate the NLRP3 inflammasome, inducing caspase-1 activation and the production of inflammatory IL-1β. Silica particles have been shown to induce caspase-1 activation and pulmonary inflammation.

In a parallel manner, a major known application of NMs is as antimicrobial agents (e.g., Ag and Cu NMs). The microbiota and immune functions are integrally linked; hence, studies should cover the impact on the interaction between bacteria and host immunity. The gut microbiome of *E. crypticus* has been shown to be altered when exposed to Cu materials^67^, shifting the communities to a decline in the relative abundance of Planctomycetes and an increase in Bacteroidetes, Firmicutes and Acidobacteria; antibiotic resistance genes in *E. crypticus* decreased significantly. The fungicide azoxystrobin also altered the abundance of core potential beneficial bacteria and increased the number and abundance of antibiotic resistance genes in the *E. crypticus* gut^68^, besides having a severe impact on survival and reproduction^69^.

#### Cytochromes P450 (CYPs)

When it comes to stress response, cytochromes P450 (CYPs), a superfamily of enzymes found across most species, are important for hormone biosynthesis and the clearance of various compounds, oxidizing steroids, fatty acids, and xenobiotics. CYPs are expanded in *E. crypticus*; their expansion in other species such as *Mesobuthus martensii* (scorpion)^70^ and *F. candida*^6^ has been associated with their survival in hazardous environments and linked to feeding on phytotoxins from herbivorous insects or larva. Hence, they could be an important mechanism of adaptation to an environment (here, soil) where toxic compounds persist and accumulate in decaying soil organic matter; these toxic compounds can include plant anti-herbivory toxins, lignocellulose breakdown products and feeding deterrents.

#### Heat shock proteins (HSPs)

The expanded HSP family are highly conserved proteins produced by cells in response to stress. For example, the HSP70 group consists of both constitutive and stress-induced HSPs as studied in *E. crypticus*^29^. One of the essential roles of HSPs under ‘normal’ conditions is to promote proper embryonic and postnatal development of multiple organ systems, particularly the nervous system^71^. A study covering embryo development and transcriptomics in response to Cd exposure showed no activation of HSPs in *E. crypticus*^22^, except for SSB1; this is in line with the findings^72^ that loss of SSB1 (combined with SSB2) impairs embryogenesis^72^. HSP70 induction as a response to stress has been shown in *E. crypticus*, for example, an increase after multigenerational exposure to copper and a turn-off when transferred to clean media^30^.

#### Purple acid phosphatases (PAPs)

PAPs are metalloenzymes that catalyze the hydrolysis of phosphomonoesters and amide substrates. PAPs are highly conserved within eukaryotic species, although varying substantially between plants and animals^73^. Functional studies indicate that PAPs have flexible mechanisms. They are well known in mammals for their involvement in bone metabolism, and functions include iron transport and generation of ROS as an immune response. For plants, a speculated function is the mobilization/scavenging of inorganic phosphate from organophosphates in the soil. The fact that the PAP family is expanded in *E. crypticus* could be related to its soil habitat and the need to extract and manage its broad range of metallic elements, as well as the response to stress. PAP seemed to be a good candidate for HGT from plants, although we did not find evidence supporting this in our analysis. For instance, in *F. candida* there was HGT from the arbuscular mycorrhizal fungus (AMF) *Rhizophagus irregularis*, which facilitates its grazing by *F. candida*^6^. In return, these AMFs benefit from spreading and inoculation to other plants, and plants benefit from phosphorus uptake from AMFs: it is a tritrophic mutualism, contributing to soil health. *F. candida* was also the first animal discovered with penicillin biosynthesis genes in its genome^6,74^; the isopenicillin N synthase gene is now also found in the *E. crypticus* HGT gene list. This suggests that these organisms have evolved to be well adapted in their soil habitat and have been able to develop antibiotic capacity in their microbe-dominated environment.

#### HAO

HAO (glycolate oxidase) 1 (HAO1) is a protein in the peroxisome encoded by the HAO1 gene in humans. HAO1 belongs to the superfamily of the alpha HAO enzymes. HAO1 catalyzes the flavin mononucleotide–mediated oxidation of glycolate to glyoxylate and glyoxylate to oxalate with reduction of oxygen to hydrogen peroxide; hence, it is central in the toxicity of ethylene glycol poisoning. The gene is primarily expressed in the liver and pancreas. Why HAO is expanded in *E. crypticus* is not obvious, but it could be for detoxifying functions, because response to stress seems to be prioritized in these organisms.

#### VTG

This is the major egg yolk precursor protein, which provides protein- and lipid-rich nutrients for developing embryos. The response of VTG to endocrine disruptive chemicals has been well studied in fish, where males can express the VTG gene in a dose-dependent manner. Invertebrates also possess an endocrine system^75^ and VTG-like proteins, although this is poorly understood. The roles of VTG and its derived yolk proteins lipovitellin and phosvitin include host innate immune defense with various functions^76^. VTG could play a role in response to stress and innate immunity in *E. crypticus*, although further studies are needed to clarify this.

#### Paramyosin

Paramyosin has been found in invertebrate muscles, and it would make sense that their expansion in *E. crypticus*, a soil worm, relates to the movement and burrowing function requirement for strong muscle contraction. Paramyosin is also a prominent antigen in human cysticercosis and may have a role as a modulator of the host immune response.

#### Potassium voltage-gated channel protein Shaw

Potassium voltage-gated channel protein Shaw, the Kv3 family, is highly represented in *E. crypticus*, and these proteins are important in shaping action potentials and in neuronal excitability and plasticity. In animal cells, the K^+^ channels are involved in neural signaling and generation of the cardiac rhythm, act as effectors in signal transduction pathways involving G protein-coupled receptors and may have a role in targeted cell lysis. Some K^+^ channels open in response to depolarization of the plasma membrane, others to hyperpolarization or an increase in intracellular calcium concentration; some can be regulated by binding of a transmitter with intracellular kinases or regulated by GTP-binding proteins or other second messengers.

#### Acetylcholine receptor

The acetylcholine (ACh) receptor (subunit beta-like 1) was well represented in *E. crypticus*. It binds ACh and responds by a change in conformation, which leads to opening of an ion-conducting channel across the plasma membrane. ACh and γ-aminobutyric acid (also present in *E. crypticus*) are among the group of neurotransmitters described in vertebrate and invertebrate nervous systems: ACh is a major excitatory transmitter, and GABA is a major inhibitory transmitter, both at the neuromuscular synapses and in the central nervous system. Several pesticides/insecticides (e.g., dimethoate) are designed to target the ACh pathway, and the impacts have been studied in regard to avoidance behavior, a relevant ecological trait for organisms to escape contaminated environments. Studies with *E. crypticus* showed an association between lack of avoidance behavior because of boric acid and an increase in the γ-aminobutyric acid receptor-associated protein, whereas acetylcholinesterase did not seem to be affected^77^. Non-avoidance to phenmedipham, however, seems to be associated with acetylcholinesterase inhibition in *E. albidus*^45,78^.

#### Inositol phosphate synthase

The expanded enzyme inositol phosphate synthase, which catalyzes the conversion of D-glucose 6-phosphate into 1D-myo-inositol 3-phosphate, is important for the production of inositol-containing compounds, including phospholipids (important for cell membrane formation and integrity), cell signaling and membrane trafficking. Mechanisms of cold adaptation or acclimation have been associated with changes in the membrane phospholipid composition, gradually undergoing a transition from liquid–crystalline to gel phase. The properties of membranes of *E. albidus* from seven populations (polar to temperate) have been studied, showing that the composition of phospholipid fatty acids varied significantly but that the ‘optimal’ fluidity of the membrane was apparently kept^79^. The accumulation of glucose, a cryoprotectant, has been observed, and glucose could possibly have a putative role in the fluidity of membranes.

#### ANK

ANK, also expanded, are a family of proteins that serve as adaptor proteins linking membrane proteins to the underlying cytoskeleton^80^. This is required to maintain the integrity of plasma membranes and to anchor specific ion channels, ion exchangers and ion transporters in the plasma membrane. Hence, both inositol phosphate synthase and ANK play a role in membrane integrity; this seems to be an important feature for *E. crypticus*, given the gene families’ expansion. In addition, ANK is required for the polarized distribution of many membrane proteins, including the Na^+^/K^+^ ATPase, the voltage-gated Na^+^ channel and the Na^+^/Ca^2+^ exchanger; hence, this must be an important regulation, because the K^+^ voltage-gated channel protein Shaw was also observed expanded. The ANK genes may have been transferred from plants and fungi to *E. crypticus* via HGT (see Supplementary Table 10, from *Phytophthora megakarya* and *Planctomycetes bacterium*). As mentioned above, ANK genes are present in an intra-scaffold palindrome, together with the protocadherin FAT4, a calcium-dependent cell adhesion protein playing a role in maintaining cell polarity. Other genes in the palindrome include serine/threonine-protein kinase pak-1 (which has important roles in cytoskeleton dynamics and cell adhesion), Ras-related protein Rab-5C (cell transporter; e.g., vesicular traffic). Hence, this hairpin gene structure with proximity between ANK, FAT4, protein kinases, etc., is not random and must aid in repairing and keeping a key function. One could argue the importance of these ionic stabilizers for their role in the observed plasticity of enchytraeids to survive in aquatic biotopes—many often living in marine interstitial environments, where the level of salts is much higher than terrestrial soil. Studies have shown that the presence of low levels of salinity (15–20‰ NaCl) clearly improves the reproduction of *E. albidus*^81^, a species often found at coastal shores among algae and in large abundances. Taking advantage of this asset from a toxicological perspective, an aquatic test has even been developed for enchytraeids^82^, and exposure to stressors in water is possible during a short period of time^83–85^, allowing researchers to screen effects via an aquatic exposure route.

### Challenges and future research applications

From the genome, a potential advance involves the possibility to confirm hypotheses and underlying mechanisms of response to stressors, often a missing link in ecotoxicology. This is feasible by using gene knock-out or gene knock-down (silencing) techniques that have been successfully demonstrated in other invertebrates, including CRISPR–Cas9^86^, transcriptional activator-like effector nucleases^87^ and RNA interference^88^. Such development and creation of proof-of-concepts will have direct impact for regulation, for example, in Registration, Evaluation, Authorisation and Restriction of Chemicals for chemicals and developing adverse outcome pathways^89^, where the causality between transcriptomics and impacts on the phenotype remains one of the main sources of uncertainty for their wider usage and implementation.

Another important future direction from the genome will be the study of the epigenome, representing one of the major regulators of observed effects and its environmental linkage. Although epigenetics has received vast attention for some species, this is not the case for invertebrates, and even less for environmentally relevant species. With the availability of the complete genome of *E. crypticus* comes the possibility of applying cheaper, more-feasible and/or more-targeted epigenetic genotyping tools, such as Methylated DNA Immunoprecipitation Sequencing (MeDIP-seq), Methyl-CpG-binding domain sequencing (MBD-seq) and epigenetic microarrays. These tools can be used to study how the genome is accessed in different cell types and during development and differentiation. These tools can also provide valuable information on how the organism is reacting on a molecular level to environmental changes. For example, we will be able to learn much more about innate immune memory and priming, information also relevant for humans.

## Conclusions

The first high-quality draft genome for *E. crypticus* was sequenced and assembled, resulting in a 525-Mbp genome, with currently >18,000 identified genes, good contiguity and completeness. Evidence suggests that *E. crypticus* may be a well-adapted species in its environment, but its genome adaptation and evolution can now be explored. Expanded gene families showed that the genome evolved to respond to stress (CYP) and to develop the innate immune system (TRIM), which are often activated via connected mechanisms. Its capacity to regenerate is a very interesting asset (LINEs), and although it has been found to be inversely related with the evolution of the innate immune system, successful regeneration requires adequate immune response. There is an obvious potential for using *E. crypticus* as a model to study interactions between regeneration, innate immune system and its aging-dependent decline. Last, the possibility of studying embryo development, a stage of high cell proliferation like regeneration, and the ability to link genes to phenotypic effects represent major advantages of working with *E. crypticus*. Available transcriptomics studies have linked response to stress to genome features. The potential for future research now includes hypothesis confirmation via gene knock-out and epigenetics.

## Methods

### Organisms

*E. crypticus* (Oligochaeta: Enchytraeidae) cultures are kept in the laboratory for many years^33^. Cultures consist of agar media plates prepared with a salt solution of CaCl_2_, MgSO_4_, KCl and NaHCO_3_, fed ad libitum with oatmeal and maintained in the laboratory under controlled conditions at 18 °C and a photoperiod of 16:8 (light:dark).

### Sample preparation and sequencing

*E. crypticus* were collected from the cultures; six pools (replicates) of 300 adult organisms (with similar size and well-developed clitellum) were rinsed in ultra-pure water and frozen in liquid nitrogen. The samples were stored at −80 °C, until further analysis.

### DNA extraction

To obtain high quality and long fragments (150 kbp) of DNA, genomic DNA was extracted from the six pools of *E. crypticus* adults by using Qiagen Blood & Cell Culture DNA Midi kit (Qiagen). Each sample was diluted in 90 µl of Tris-EDTA (TE) buffer, pH 8.0. The quantity and purity of the isolated DNA were measured spectrophotometrically with a nanodrop (NanoDrop ND-1000 spectrophotometer), and its quality and fragment sizes were analyzed on 1% weight/volume (w/v) agarose gel, by pulse-field gel electrophoresis (CHEF-DR II pulse-field electrophoresis system). After the confirmation of DNA quality, the six samples of *E. crypticus* were pooled.

### Short-read library preparations and sequencing

High-molecular-weight genomic DNA of *E. crypticus* was quantified by using picogreen (Invitrogen), and its integrity was checked on a 1% (w/v) E-gel (Invitrogen). For the construction of a paired-end library, 2.5 µg of genomic DNA was sheared by using a Covaris S2 sonicator (Covaris), aiming for 500-bp fragments. The fragmented DNA was used to build a PCR-free sequencing library with the NEBNext Ultra II DNA library prep kit for Illumina (New England BioLabs) by using TruSeq adapters. For the construction of a mate-pair library, 1 µg of intact genomic DNA was used with the Nextera mate pair library prep kit (Illumina). Both sequencing libraries were size-selected on a 2% (w/v) agarose E-gel. Fragments ranging from 700 to 800 bp were cut out of the gel and purified with the Zymoclean gel recovery kit (Zymo Research). Library quality was checked on an Agilent Bioanalyzer by using a high-sensitivity chip (Agilent Technologies), and concentration was measured via qPCR according to the qPCR Quantification Protocol (Illumina). Paired-end and mate-pair libraries were pooled with a 75:25 percent ratio and sequenced for 2 × 150 cycles in two lanes on a HiSeq3000 (Illumina).

### Long-read sequencing library preparations and sequencing

High-molecular-weight DNA (no shearing required) was purified with 0.6× AMPure PB beads (Beckman Coulter) and eluted in Elution Buffer (EB) buffer. Quantification was done on a Qubit (Thermo Fisher Scientific) by using the ‘Broad Range Kit’. A sequencing library was prepared by using the SMRTbell express template prep kit (PacBio) according to the ‘Procedure & Checklist - Preparing > 15 kb Libraries Using SMRTBell Template Preparation Kit’ (PacBio). Library fragments in the 15–50-kb range were size-selected on a Blue Pippin (Sage Science) by using a 0.75% dye-free agarose gel cassette according to the ‘0.75% DF Marker S1 high-pass 15–20 kb protocol’. Library quality was checked on a Fragment Analyzer (Agilent) by using the ‘High Sensitivity Large Fragment 50 kb Kit’. Final yield was measured on a Qubit by using the ‘High Sensitivity Kit’. The library was sequenced on a PacBio Sequel I (PacBio) by using the V2.1 Binding kit, Chemistry kit and three SMRT cells. The loading type was ‘diffusion’ with an on-plate concentration of 6 pM. There was no pre-extension, and data were collected as a 10-h movie.

### De novo genome assembly

For Illumina mate-pair reads, junction adapter removal and read categorization were done by using nxtrim (v0.4.3, Illumina). The resulting true mate-pair reads were used in combination with unmodified Illumina paired-end reads and PacBio long reads for a hybrid de novo genome assembly with MaSuRCA (v3.2.8)^90^ by using default settings. The quality and integrity of the assembly were assessed with Quast (v4.6.2)^91^ and BUSCO (v4.0.6)^92^.

The mitochondrial genome was assembled separately by using the Illumina paired-end reads with NOVOPlasty (v2.7.2)^93^. Scaffolds in the genome assembly consisting of fragments of the mitochondrial genome were removed.

All sequencing reads and the *E. crypticus* genome (including the mitochondrial genome) were submitted at the European Nucleotide Archive as study PRJEB41884. The de novo genome assembly is available via accession number GCA_905160935.

### Genome annotation

Annotation of the assembled genome was done iteratively by using Maker (v2.31.10)^94^. Repetitive sequences were masked with RepeatMasker (v4.0.7) and RepeatModeler (v1.0.11)^95,96^. A first round of annotation with biological evidence was done by using publicly available transcriptome data from *E. crypticus* (National Center for Biotechnology Information accession GALF01), cDNA data and protein sequences from the related organism *E. albidus* collected from the National Center for Biotechnology Information (NCBI), as well as all proteins from Uniprot/Swissprot. Next, several ab initio gene predictors were trained and applied in succession to improve the accuracy of identified features on the genome: two training and application rounds of SNAP (v2006–07–28)^97^ and a single training and application round of Augustus (v3.2.3)^98^ and GeneMark (v4.38)^99^. Transfer RNA genes were identified by using trnaScan-SE (v1.3.1)^100^, and ribosomal RNA genes were detected by using barrnap (v0.9)^101^. Protein domain information and GO terms were added by using Interproscan (v5.29)^102^. Finally, a putative function was assigned to the predicted genes by blastp comparison (E-value threshold: 10^−6^) of the derived protein sequences with the Uniprot/Swissprot database. The mitochondrial genome was annotated by using MITOS (v1.0.1)^103^. For details on the bioinformatics settings, please see Supplementary Table 12.

### Gene family analysis and orthogroups

Identification of homologous protein coding genes between *E. crypticus* and eight other relevant species was performed by using the protein sequences of the selected species and OrthoFinder (v2.4.0), with default settings to infer orthogroups^104^. These groups contain all genes descending from a single gene in the last common ancestor of the species whose genes are being analyzed. The eight species (Supplementary Table 13) included in the comparison were selected for being relevant models (*C. elegans* (Nematoda) and *D. melanogaster* (Insecta)), ecotoxicology models (*F. candida* (Collembola)^6^ and *D. pulex* (Crustacea)^8^) and phylogenetically closely related annelids (*C. teleta* (Polychaete, a marine polichaete), *H. robusta* (Clitellata, Hirudinida, a freshwater leech), *E. andrei* (Clitellata) and *E. fetida* (Clitellata)^105^). *M. solifugus*, a glacier enchytraeid species^9^, could not be included because its genome sequence information is not available^106^.

To identify significant expansions and contractions of gene families in *E. crypticus*, the z-score method^70^ was used. For each gene family (orthogroup), represented by at least three species besides *E. crypticus*, a z-score was calculated for each species as: (the gene number for the species – the average gene number of the family from all species)/the standard deviation of gene numbers of the family from all species. The families with z-scores ≥2 were considered as significantly expanded, whereas families with a z-score ≤−2 were considered contracted.

A phylogenetic tree was built on the basis of the orthogroup analysis, and the age of the root was derived from the TimeTree database^50^.

To identify lineage-specific gene families of *E. crypticus* among this group of eight species, the orthogroups that contain zero genes in all but *E. crypticus* were filtered out.

### Collinearity analysis

Intragenomic collinearity and synteny of the *E. crypticus* genes were investigated by using MCScanX^107^.

### HGT

HGT analysis was performed as described in ref. ^6^. In short, all *E. crypticus* transcripts were blasted against (i) a protein database containing all metazoan proteins (excluding annelida) and (ii) a protein database containing all non-metazoan proteins. All protein sequence databases were downloaded from Uniprot. Then, an h score was calculated as the best non-metazoan blast hit bitscore – the best metazoan blast hit bitscore. A gene with an h score ≥30 and best non-metazoan bitscore ≥100 is considered an HGT candidate. Furthermore, the HGT candidate gene under investigation must have native genes on the same scaffold, and a linkage to neighboring native genes must be confirmed by the presence of mapped PacBio long reads. When linkage and neighbor requirements were fulfilled, candidates were considered confirmed as HGT when the best metazoan bitscore was <50. When linkage was fulfilled but the best metazoan bitscore was ≥50, we performed a phylogenetic validation. To this end, the HGT candidate was blasted against several protein databases: (i) metazoa excluding annelida, (ii) annelida, (iii) plants, (iv) bacteria, (v) archaea and (vi) protists. For each HGT candidate, the top five blast hits in each database were collected and aligned with the HGT by using muscle (v3.8.31). The sequence alignment was trimmed with trimal (v1.4), and a phylogenetic tree was derived by using phyml (v3.3) with the approximate likelihood ratio test (aLRT) method. The trees were inspected, and the HGT candidate was confirmed when the smallest clade containing the HGT candidate did not contain any metazoan sequences.

### Hox genes

To identify and assess the organization of Hox genes, we compared the complete HomeoDB^108^ set of homeodomain proteins to the *E. crypticus* protein-coding genes via blastp.

### Reporting Summary

Further information on research design is available in the Nature Research Reporting Summary linked to this article.

## Online content

Any methods, additional references, Nature Research reporting summaries, source data, extended data, supplementary information, acknowledgements, peer review information; details of author contributions and competing interests; and statements of data and code availability are available at 10.1038/s41684-021-00831-x.

## Supplementary information

Supplementary Information Supplementary Figures 1–3

Reporting Summary

Supplementary Tables 1–13 Excel workbook with 13 sheets of supplemental tables

Supplementary Data Genome file

## Data Availability

All data are available and will be provided upon request if necessary. The de novo genome assembly is available via accession number GCA_905160935.
