# Supplementary material for: Annelid genomes: Enchytraeus crypticus, a soil model for the innate (and primed) immune system
Source: Lab Anim (NY). 2021 Sep 6;50(10):285–94. doi: 10.1038/s41684-021-00831-x (PMC8460440; doi:10.1038/s41684-021-00831-x)
Supplement: Supplementary file 1 — Supplementary Figures 1–3 [file 41684_2021_831_MOESM1_ESM.pdf]

nature portfolio

https://doi.org/10.1038/s41684-021-00831-x

Supplementary information

Annelid genomes: *Enchytraeus crypticus*, a soil model for the innate (and primed) immune system

In the format provided by the authors and unedited

Supplementary figures

Annelid genomes: *Enchytraeus crypticus*, a soil model for innate (and primed) immune system

Mónica J.B. Amorim[1*], Yannick Gansemans[2], Susana I.L. Gomes[1], Filip Van Nieuwerburgh[2] and Janeck J. Scott-Fordsmand[3]

[1] Department of Biology & CESAM, University of Aveiro, 3810-193 Aveiro, Portugal

[2] Department of Pharmaceutics, Laboratory of Pharmaceutical Biotechnology, Ghent University, 9000 Ghent, Belgium

[3] Department of Biosciences, Aarhus University, 8600 Silkeborg, Denmark

* corresponding author: mjamorim@ua.pt

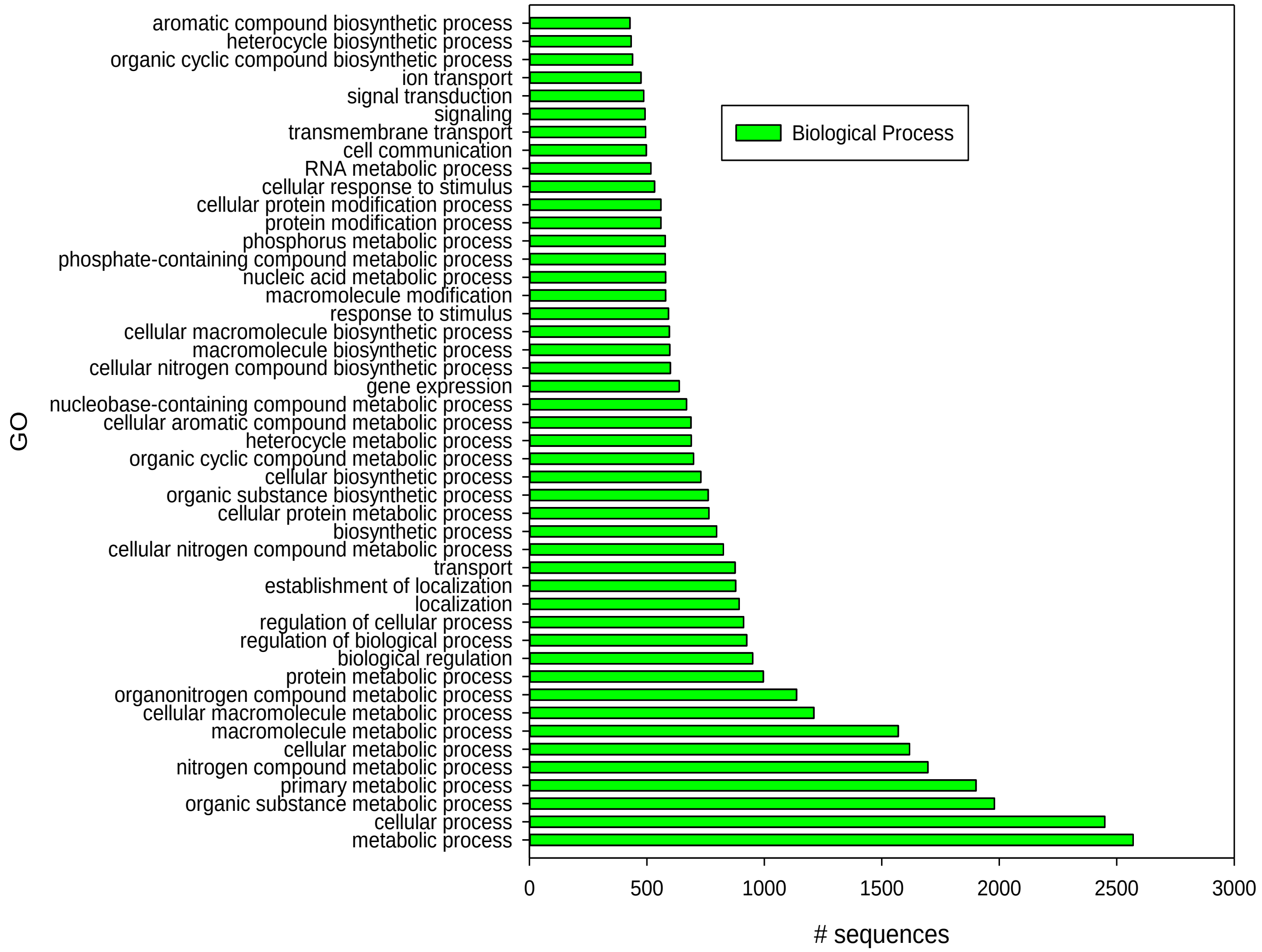

Biological Process
aromatic compound biosynthetic process
heterocycle biosynthetic process
organic cyclic compound biosynthetic process
ion transport
signal transduction
signaling
transmembrane transport
cell communication
RNA metabolic process
cellular response to stimulus
cellular protein modification process
protein modification process
phosphorus metabolic process
phosphate-containing compound metabolic process
nucleic acid metabolic process
macromolecule modification
response to stimulus
cellular macromolecule biosynthetic process
macromolecule biosynthetic process
cellular nitrogen compound biosynthetic process
gene expression
nucleobase-containing compound metabolic process
cellular aromatic compound metabolic process
heterocycle metabolic process
organic cyclic compound metabolic process
cellular biosynthetic process
organic substance biosynthetic process
cellular protein metabolic process
biosynthetic process
cellular nitrogen compound metabolic process
transport
establishment of localization
localization
regulation of cellular process
regulation of biological process
biological regulation
protein metabolic process
organonitrogen compound metabolic process
cellular macromolecule metabolic process
macromolecule metabolic process
cellular metabolic process
nitrogen compound metabolic process
primary metabolic process
organic substance metabolic process
cellular process
metabolic process
GO
0
500
1000
1500
2000
2500
3000
sequences

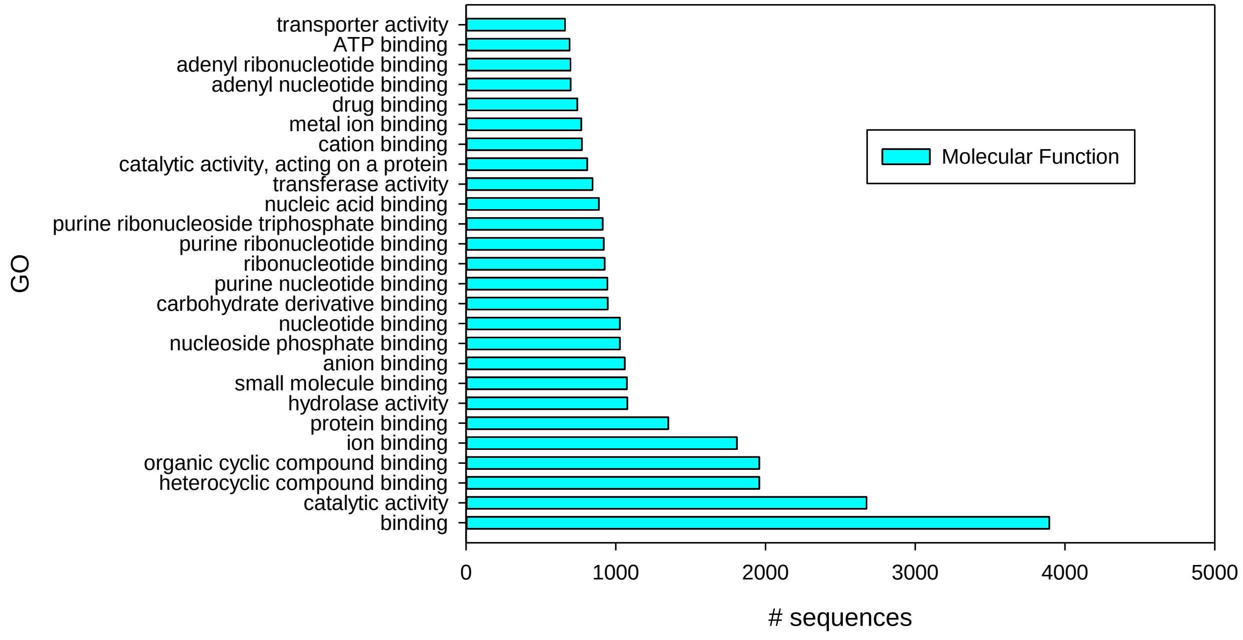

Molecular Function
transporter activity
ATP binding
adenyl ribonucleotide binding
adenyl nucleotide binding
drug binding
metal ion binding
cation binding
catalytic activity, acting on a protein
transferase activity
nucleic acid binding
purine ribonucleoside triphosphate binding
purine ribonucleotide binding
ribonucleotide binding
purine nucleotide binding
carbohydrate derivative binding
nucleotide binding
nucleoside phosphate binding
anion binding
small molecule binding
hydrolase activity
protein binding
ion binding
organic cyclic compound binding
heterocyclic compound binding
catalytic activity
binding
GO
0
1000
2000
3000
4000
5000
sequences

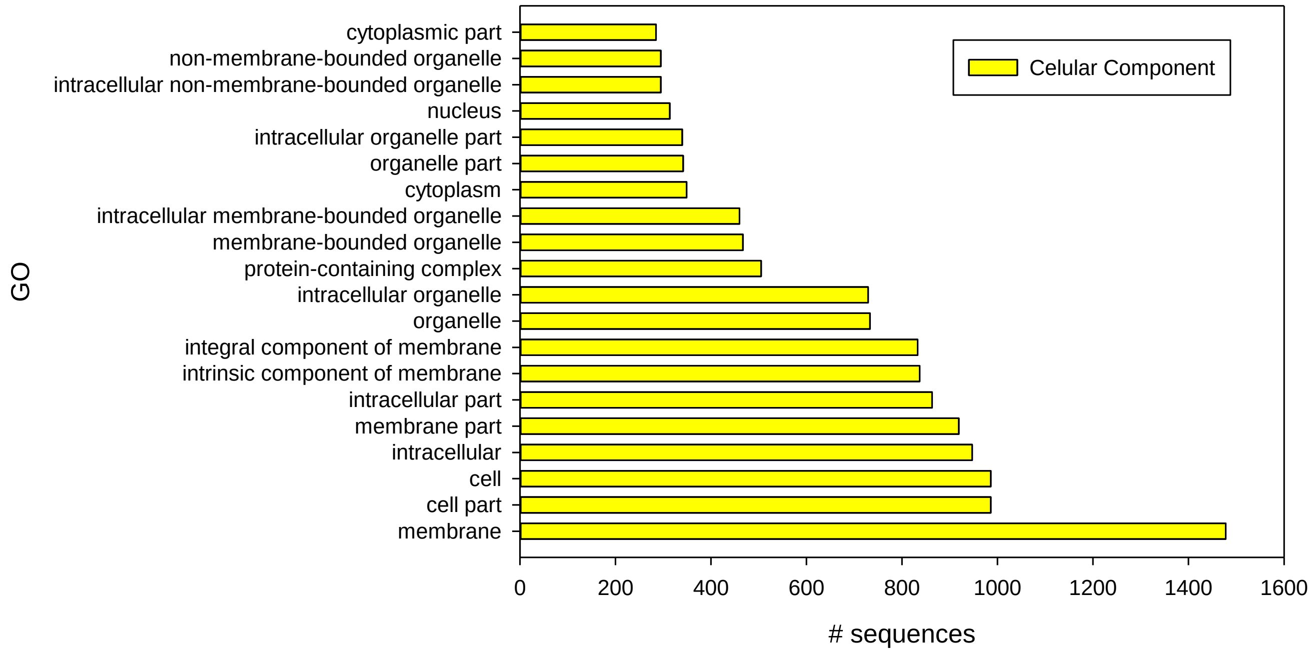

Figure S1: Distribution of GO terms assigned to the 16,424 annotated sequences.

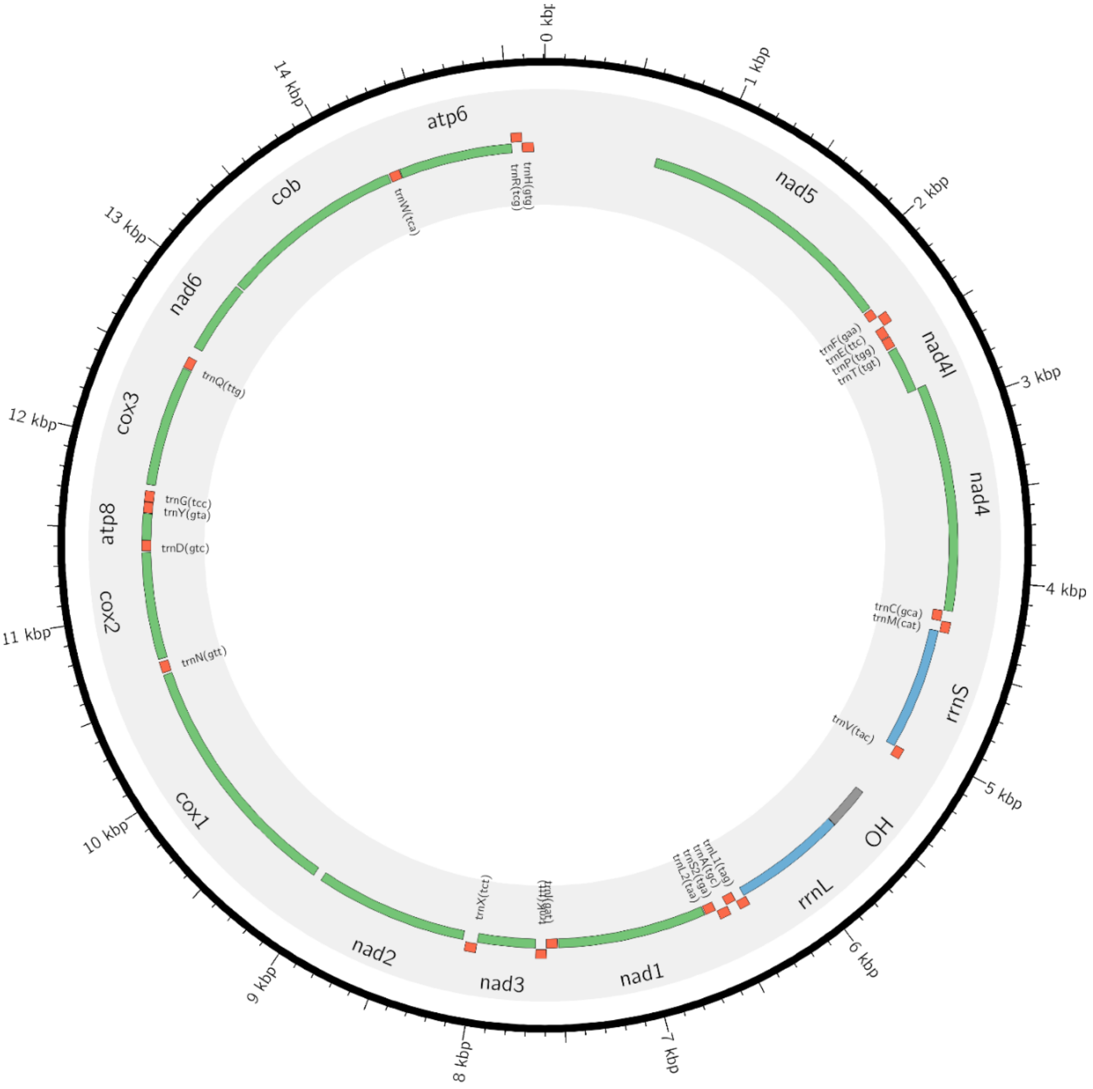

Figure S2: Map of the 15,205 bp *Enchytraeus crypticus* mitochondrial genome.

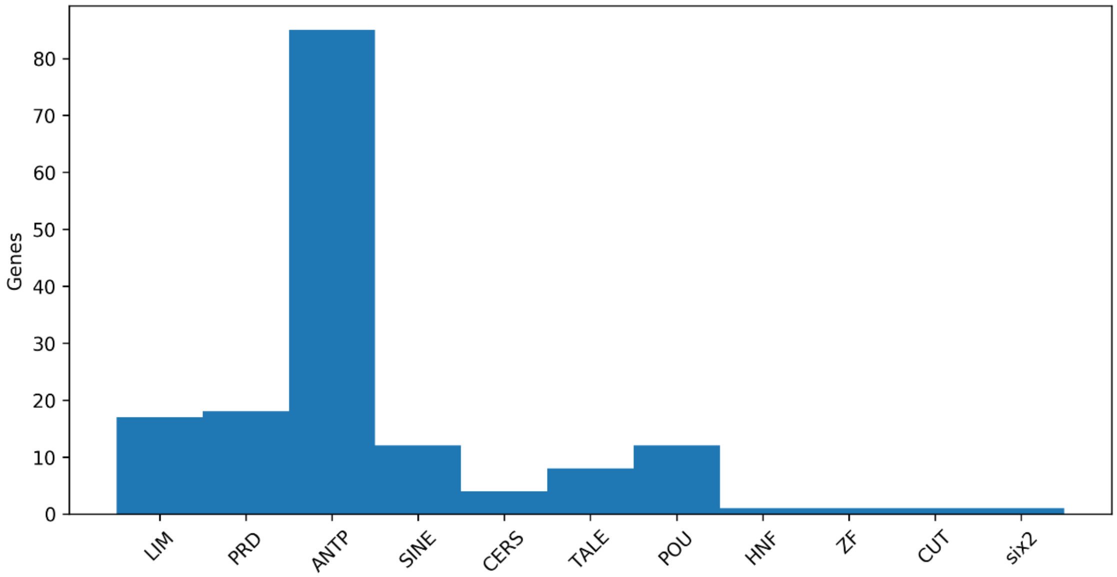

Figure S3: *Enchytraeus crypticus* gene counts per homeobox class. Class names are according to the HomeoDB nomenclature.
